# Improving the use of research evidence in guideline development: 12. Incorporating considerations of equity

**DOI:** 10.1186/1478-4505-4-24

**Published:** 2006-12-05

**Authors:** Andrew D Oxman, Holger J Schünemann, Atle Fretheim

**Affiliations:** 1 Norwegian Knowledge Centre for the Health Services, P.O. Box 7004, St. Olavs plass, N-0130 Oslo, Norway; 2 INFORMA, S.C. Epidemiologia, Istitituto Regina Elena, Via Elio Chianesi 53, 00144 Rome, Italy

## Abstract

**Background:**

The World Health Organization (WHO), like many other organisations around the world, has recognised the need to use more rigorous processes to ensure that health care recommendations are informed by the best available research evidence. This is the 12^th ^of a series of 16 reviews that have been prepared as background for advice from the WHO Advisory Committee on Health Research to WHO on how to achieve this.

**Objectives:**

We reviewed the literature on incorporating considerations of equity in guidelines and recommendations.

**Methods:**

We searched PubMed and three databases of methodological studies for existing systematic reviews and relevant methodological research. We did not conduct systematic reviews ourselves. Our conclusions are based on the available evidence, consideration of what WHO and other organisations are doing and logical arguments.

**Key questions and answers:**

We found few directly relevant empirical methodological studies. These answers are based largely on logical arguments.

When and how should inequities be addressed in systematic reviews that are used as background documents for recommendations?

• The following question should routinely be considered: Are there plausible reasons for anticipating differential relative effects across disadvantaged and advantaged populations?

• If there are plausible reasons for anticipating differential effects, additional evidence should be included in a review to inform judgments about the likelihood of differential effects.

What questions about equity should routinely be addressed by those making recommendations on behalf of WHO?

• The following additional questions should routinely be considered:

• How likely is it that the results of available research are applicable to disadvantaged populations and settings?

• How likely are differences in baseline risk that would result in differential absolute effects across disadvantaged and advantaged populations?

• How likely is it that there are important differences in trade-offs between the expected benefits and harms across disadvantaged and advantaged populations?

• Are there different implications for disadvantaged and advantaged populations, or implications for addressing inequities?

What context specific information is needed to inform adaptation and decision making in a specific setting with regard to impacts on equity?

• Those making recommendations on behalf of WHO should routinely consider and offer advice about the importance of the following types of context specific data that might be needed to inform adaptation and decision making in a specific setting:

• Effect modifiers for disadvantaged populations and for the likelihood of differential effects

• Baseline risk in relationship to social and economic status

• Utilization and access to care in relationship to social and economic status

• Costs in relationship to social and economic status

• Ethics and laws that may impact on strategies for addressing inequities

• Availability of resources to address inequities

What implementation strategies are likely be needed to ensure that recommendations are implemented equitably?

• Organisational changes are likely to be important to address inequities. While it may only be possible to consider these in relationship to specific settings, consideration should be given to how best to provide support for identifying and addressing needs for organisational changes. In countries with pervasive inequities institutional, cultural and political changes may first be needed.

• Appropriate indicators of social and economic status should be used to monitor the effects of implementing recommendations on disadvantaged populations and on changes in social and economic status.

## Background

The World Health Organization (WHO), like many other organisations around the world, has recognised the need to use more rigorous processes to ensure that health care recommendations are informed by the best available research evidence. This is the 12^th ^of a series of 16 reviews that have been prepared as background for advice from the WHO Advisory Committee on Health Research to WHO on how to achieve this.

Braveman and Gruskin define equity as "the absence of disparities in health that are systematically associated with social advantage or disadvantage" [1]. The message is made clearer by Margaret Whitehead's definition of inequity: "differences in health which are not only unnecessary and avoidable but, in addition, are considered unfair and unjust" [2]. Inequities in health and health care are well documented in relationship to social and economic factors, including Place of residence (e.g. rural, urban, inner city, Race/ethnicity/culture, Occupation, Gender, Religion, Educational level, Socioeconomic status and Social capital (availability of neighbourhood support, social stigma, civic society) (PROGRESS) [3].

Disadvantaged populations almost always have poorer health [4], poorer access to health care [5], and receive poorer quality health care [6]. To the extent that recommendations influence what is done, they can improve the overall health of the population but have no impact on inequities, reduce inequities or exacerbate them regardless of the overall effects on population health. There has been a growing interest in taking equity into consideration in clinical practice guidelines [7,8]. However, consideration of inequities has generally been lacking [7]. For example, AGREE and other instruments for assessing the quality of guidelines do not include items on equity or the fairness of the recommendations [9].

In this paper we address the following questions:

• When and how should inequities be addressed in systematic reviews that are used as background documents for recommendations?

• What questions about equity should routinely be addressed by those making recommendations on behalf of WHO?

• What context specific information is needed to inform adaptation and decision making in a specific setting with regard to impacts on equity?

Related questions about adaptation, applicability and transferability are addressed in another paper in this series [10].

## What WHO is doing now

"WHO has embraced the elimination of health inequities as an important target and supports the dual goals of equity and efficiency for health services. WHO's data gathering on inequalities in health status and access to services is shaped by and in turn informs its advocacy and normative activities that aim to reduce health inequities. Besides collecting relevant data broken down by group, WHO attempts both to relate these data to health determinants (e.g., membership in less privileged social groups and exposure to various hazards) and to develop and disseminate interventions to improve conditions for members of such groups" [11].

Nonetheless, we are not aware of any specific documents that provide guidance as to how equity should be taken into account in WHO guidelines or recommendations or of any studies or descriptions of current practice. The WHO guidelines for guidelines do not currently provide any explicit advice regarding how to take account of equity.

## What other organisations are doing

Clinical practice guidelines typically focus on the effectiveness of interventions (Will adherence to a recommendation do more good than harm?), occasionally on cost-effectiveness (Are the net benefits worth the costs?), and rarely on equity (Are the recommendations fair?) [7]. More recently, several guideline developers have begun to consider equity explicitly and systematically, including, for example, the Australian NHMRC [7], INCLEN [8], the GRADE Working Group, and the National Institute for Health and Clinical Excellence (NICE) in the UK, which now has an extended mandate including public health guidance and reducing health inequalities, after the Health Development Agency (HDA) became part of NICE in 2005 [12]. The HDA was established in 2000 to develop the evidence base to improve health and reduce health inequalities. It worked in partnership with professionals and practitioners across a range of sectors to translate that evidence into practice. Other countries that have had a major political commitment to reducing inequities in health include the Netherlands [13], Thailand, and Chile [14].

## Methods

The methods used to prepare this review are described in the introduction to this series [15]. Briefly, the key questions addressed in this paper were vetted amongst the authors and the ACHR Subcommittee on the Use of Research Evidence (SURE). We did not conduct a full systematic review. We searched PubMed and three databases of methodological studies (the Cochrane Methodology Register [16], the US National Guideline Clearinghouse [17], and the Guidelines International Network [18]) for existing systematic reviews and relevant methodological research that address these questions. We did not conduct systematic reviews ourselves. The answers to the questions are our conclusions based on the available evidence, consideration of what WHO and other organisations are doing, and logical arguments.

This paper is based in large part on a workshop on addressing inequities held in Oslo August 31 to September 1, 2005 [19], background documentation for that workshop [20-23], and a reference list generated during and subsequent to the workshop. We searched PubMed using (clinical practice guidelines or public health guidelines) and (equity or equality) and related articles for references [7] and [23]. We searched the Cochrane Methodology Register using equity or equality.

## Findings

Our database searches yielded few references and we found few directly relevant empirical methodological studies, consistent with the findings of other reviews [22,23]. For example, the literature search and correspondence with guideline developers worldwide by the NHMRC located no examples of where clinical practice guideline developers explicitly incorporated evidence on socioeconomic position and health into generic guidelines, except for when guidelines were developed for specific disadvantaged sub-populations [22]. This is consistent with the findings of the Health Development Agency in England. They observed that there is a very large literature that describes the problem of inequalities and a very much smaller one describing interventions that could reduce inequalities [24].

### When and how should inequities be addressed in systematic reviews that are used as background documents for recommendations?

Evidence of the effects of interventions on inequities is sparse and difficult to search for [25]. For example, Tsikata and colleagues found that only 10% of controlled trials assessed the efficacy of the intervention across socioeconomic subgroups [26]. Similarly, Ogilvie and colleagues found that in Cochrane reviews of controlled studies of tobacco control both the reviews and the primary studies in those reviews rarely assessed the impact of the intervention across socioeconomic factors [27]. Systematic reviews tend not to provide evidence on differential effectiveness [27-33]. Searches of electronic databases in many fields, particularly for social interventions and more upstream interventions, may miss much relevant evidence [31-33]. Publication bias may be a problem [25]. Because there is limited direct evidence of differential effects of interventions across socioeconomic groups, it will generally be necessary to search for and include a wider scope of evidence to support or refute plausible hypotheses of differential effects, or the effects of interventions on reducing inequities.

Although there are clear arguments for exploring moderator effects in systematic reviews, subgroup analyses can be misleading both because of inadequate power (resulting in false negative conclusions) and multiple testing (resulting in false positive conclusions) [34-38]. The results observed in subgroups may differ by chance from the overall effect identified by the meta-analysis, and the subgroup findings may not be confirmed by subsequent large trials [36,39]. Paradoxically, the best estimate of the outcome of the intervention in a sub-group may come from discounting the results of the sub-group analysis and using the overall results (Stein's paradox) [36,40]. General guidelines for interpreting subgroup analyses can be applied to subgroup analyses based on socioeconomic factors [40,41].

### What questions about equity should routinely be addressed by those making recommendations on behalf of WHO?

Additional questions that should be considered in relationship to equity include questions about the applicability of the evidence to disadvantaged populations, differences in values, and the implications of these differences. General guidelines for considering the applicability of evidence can be applied to considering the applicability of evidence to disadvantaged populations [42], including differences in absolute effects due to differences in baseline risk. The trade-offs between the benefits and harms of an intervention may be different because of differences in the relative or absolute effects of an intervention or because of differences in values [8]. For example, if an outcome, such as the ability to quickly return to or stay at work, is more important to disadvantaged populations, this might tip the balance between the benefits, harms and costs of an intervention (for example antiretrovirals for AIDS) in favour of intervening. Differences in any of these factors can result in different implications and recommendations for disadvantaged populations or specific recommendations for addressing inequities [8].

### What context specific information is needed to inform adaptation and decision making in a specific setting with regard to impacts on equity?

While evidence about the effects of interventions generally comes from global research, it is necessary to take into account factors in a specific setting to inform decisions about what to do. These factors include each of the following in relationship to socioeconomic factors: the presence of effect modifiers that have been identified in the global research, baseline risk, utilization and access to care, and costs. In addition, it is necessary to take into account relevant ethical and legal standards in a specific setting, and the availability of resources to address inequities. Although this information is beyond the scope of a review or international guidelines or recommendations, international groups can systematically consider the need for these different types of information in specific settings and provide guidance regarding the importance of obtaining such information and practical strategies for doing so and integrating context specific information into decision-making processes.

### What implementation strategies are likely be needed to ensure that recommendations are implemented equitably?

Because disadvantaged populations generally have poorer access to care and often receive poorer quality care, organisational changes are likely to be needed to address inequities in health care. Organisational changes are also likely to be necessary to implement interventions targeted at social determinants of health. Identifying necessary organisational changes, and barriers and facilitators of implementing change requires context specific knowledge and decisions. Nonetheless, general guidance and support for what information to consider, possible strategies to address common barriers and facilitators, and general frameworks for planning organisational changes and implementation strategies can be provided internationally. In countries with pervasive inequities institutional, cultural and political changes may first be needed.

Similarly, although local data are needed to monitor the effects of implementing recommendations, guidance can be provided regarding appropriate indicators of social gradients and measures of change (e.g. in the ratio of quintile 1 to 5, or concentration indices) to use in order to monitor the effects of implementing recommendations on disadvantaged populations and on changes in social gradients. Because the evidence for interventions to reduce inequities will commonly be weak, it is generally important to ensure that monitoring and evaluations are as rigorous as possible to ensure that intended effects are achieved and unintended adverse effects are avoided.

### What 'maps' are available of the different dimensions of inequity locally?

Equity and inequity are not one-dimensional phenomena. They consist of a number of dimensions that include economic status, occupation, gender, ethnicity, class, caste, religion, status grouping, age, disability, place of residence, geographical location, and manifest sexual orientation. These different dimensions are of varying salience in any given social context. For example caste and religion are more frequently significant in pre industrial systems while occupation tends to be dominant in industrial systems. It is also important to note that the importance of these various dimensions relative to each other also varies, as the dimensions overlap and overlay each other. The health effects of inequities are a product of the interplay of these different dimensions. It is therefore important to describe systematically the dimensions, and if possible their relative salience, in any given social arrangement.

## Discussion

Inequities are rarely addressed in clinical practice guidelines. Evidence of the effects of public health and health policy interventions on reducing inequities is generally weak or lacking [43]. As a consequence, advice regarding how to address inequities in recommendations must to a large extent rely on the application of general methodological studies and principles, for example in relationship to subgroup analyses and applicability. While addressing inequities is a fundamental concern at the heart of WHO's mission, at present there appears to be inadequate guidance on how best to do this in developing and implementing recommendations.

Although we have not found empirical descriptions of WHO's current practices, it is reasonable to assume that inequities are not being addressed systematically and transparently. This assumption rests in part on documentation that WHO guidelines generally have not adhered to standards such as AGREE [44,45]. WHO may be more likely to address inequities than many other organisations, given its mission. However, the available evidence suggests that inequities are generally not well addressed in most systematic reviews and clinical practice guidelines. It is only recently that attention has been given to the methods used to address inequities, both for clinical and public health interventions [7,21,46].

## Further work

Although we have not conducted a systematic review of the relevant literature, a more systematic review is not likely to have results or implications that are substantially different, given the sparseness of methodological research in this area. This assumption is supported by the NHMRC review [7] and a NHS HTA review of addressing equity in economic analyses [23]. However, growing attention is being paid to this area and there are areas of research that can further inform specific issues, such as the selection of indicators of socioeconomic status in relationship to specific interventions or conditions. Thus, while we do not believe that WHO should undertake further work at this time, it would be valuable for WHO or others to undertake and keep up-to-date systematic methodology reviews that address specific aspects of how to address inequities in systematic reviews, guidelines and recommendations.

## Competing interests

ADO and AF work for the Norwegian Knowledge Centre forthe Health Services, an agency funded by the Norwegian government that produces systematic reviews and health technology assessments. All three authors are contributors to the Cochrane Collaboration. ADO and HJS are members of the GRADE Working Group. HJS is documents editor and chair of the documents development and implementation committee for the American Thoracic Society and senior editor of the American College of Chest Physicians' Antithrombotic and Thrombolytic Therapy Guidelines.

## Authors' contributions

ADO prepared the first draft of this review. HJS and AF contributed to drafting and revising it.
